# Regulation of Muscle Glycogen Metabolism during Exercise: Implications for Endurance Performance and Training Adaptations

**DOI:** 10.3390/nu10030298

**Published:** 2018-03-02

**Authors:** Mark A. Hearris, Kelly M. Hammond, J. Marc Fell, James P. Morton

**Affiliations:** Research Institute for Sport & Exercise Sciences, Liverpool John Moores University, Liverpool L3 3AF, UK; M.Hearris@2014.ljmu.ac.uk (M.A.H.); K.M.Hammond@2009.ljmu.ac.uk (K.M.H.); J.M.Fell@2015.ljmu.ac.uk (J.M.F.)

**Keywords:** muscle glycogen, glucose, athletes, train-low

## Abstract

Since the introduction of the muscle biopsy technique in the late 1960s, our understanding of the regulation of muscle glycogen storage and metabolism has advanced considerably. Muscle glycogenolysis and rates of carbohydrate (CHO) oxidation are affected by factors such as exercise intensity, duration, training status and substrate availability. Such changes to the global exercise stimulus exert regulatory effects on key enzymes and transport proteins via both hormonal control and local allosteric regulation. Given the well-documented effects of high CHO availability on promoting exercise performance, elite endurance athletes are typically advised to ensure high CHO availability before, during and after high-intensity training sessions or competition. Nonetheless, in recognition that the glycogen granule is more than a simple fuel store, it is now also accepted that glycogen is a potent regulator of the molecular cell signaling pathways that regulate the oxidative phenotype. Accordingly, the concept of deliberately training with low CHO availability has now gained increased popularity amongst athletic circles. In this review, we present an overview of the regulatory control of CHO metabolism during exercise (with a specific emphasis on muscle glycogen utilization) in order to discuss the effects of both high and low CHO availability on modulating exercise performance and training adaptations, respectively.

## 1. Introduction

The study of carbohydrate (CHO) metabolism in relation to the field of sport and exercise is an area of investigation that is now over 100 years old. Almost a century ago, Krogh and Lindhard [1] reported the efficiency of CHO as a fuel source during exercise and demonstrated that fatigue occurs earlier when subjects consume a low CHO diet (as compared with a high CHO diet) in the days preceding an exercise bout undertaken at a fixed workload. Levine et al. [2] also observed that runners who completed the 1923 Boston marathon exhibited hypoglycemia (<4 mmol·L^−1^) immediately post-exercise, thus suggesting that low CHO availability may be linked to fatigue. These early studies provided the initial evidence that CHO was an important fuel source for sustaining exercise performance. 

Nonetheless, much of the foundation of our understanding of CHO metabolism was developed by Scandinavian researchers in the late 1960s with the introduction of the muscle biopsy technique [3,4,5,6]. These researchers provided the platform for modern day sports nutrition practice in a series of studies that collectively demonstrated that (1) muscle glycogen is depleted during exercise in an intensity dependent manner; (2) high CHO diets increase muscle glycogen storage and subsequently improve exercise capacity and (3) muscle glycogen storage is acutely enhanced following prior glycogen depletion (i.e., the super-compensation effect), the magnitude of which is dependent on high CHO availability [4]. This body of work remains some of the most highly cited papers in the field and is referenced accordingly in the most recent sport nutrition guidelines [7]. 

The field continued to develop throughout the 1980s and 1990s with the consistent finding that CHO feeding during exercise also improved exercise performance and capacity [8,9,10,11,12]. Such studies relied on the use of stable isotope methodology (to quantify exogenous CHO oxidation) as well as magnetic resonance imaging to quantify liver glycogen depletion during exercise [13]. As such, it is now generally accepted that liver glycogen depletion is also a major contributing cause of fatigue during endurance exercise. It is noteworthy, however, that CHO feeding can also improve physical performance via non-metabolic effects through modulating regions of the brain associated with reward and motor control [14,15].

In addition to a simple “fuel store”, our understanding of CHO metabolism has advanced considerably in recent years with the use of more sophisticated molecular biology techniques. In this regard, it is now accepted that glycogen is more than a store [16], acting as a regulator of many key cell signaling pathways related to promoting the oxidative phenotype, insulin sensitivity, contractile processes, protein degradation and autophagic processes [16,17]. When considered this way, it is remarkable that whole body storage of only 500 g of substrate can exert such profound effects on multiple tissues, organs and systems, the results of which have considerable effects on human health and performance. 

The aim of this review is to, therefore, present a contemporary discussion of our understanding of CHO metabolism (focusing on muscle glycogen metabolism) with specific reference to sport and exercise. We begin by presenting an overview of CHO storage followed by outlining regulatory steps in the control of both muscle glycogen metabolism and muscle glucose uptake. We then proceed to discuss how manipulating substrate availability (i.e., CHO availability itself) and alterations to specifics of the exercise protocol (e.g., intensity, duration) and training status of the athlete can all affect the magnitude of CHO utilized during exercise. The previous section, therefore, provides the platform to discuss the well-documented effects of both endogenous (i.e., liver and muscle glycogen) and exogenous (i.e., CHO feeding during exercise) CHO availability on exercise performance. Finally, we close by discussing the role of CHO availability on modulating aspects of training adaptation, a field of research that has grown rapidly in the last decade.

## 2. Overview of CHO Storage

**Figure 1:**
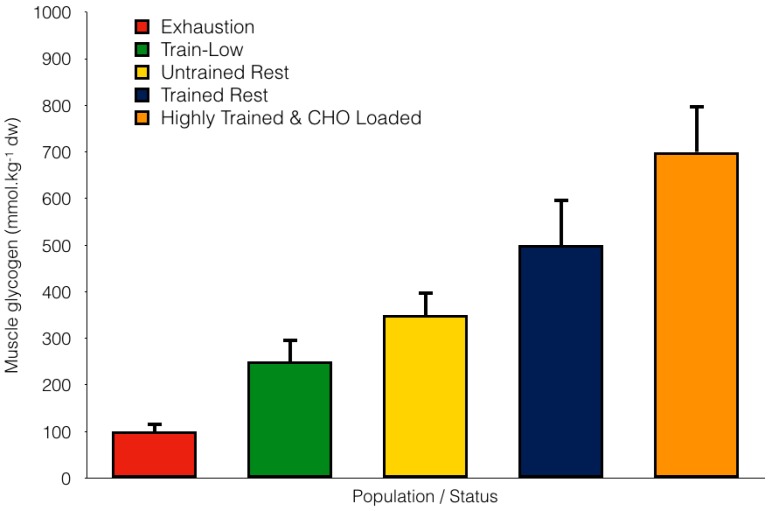
Variations in muscle glycogen storage according to fatigue status, training status and dietary carbohydrate (CHO) intake (data are compiled from males only and from several studies including Taylor et al. [30]; Bartlett et al. [31]; Arkinstall et al. [32]; Gollnick et al. [24]; Coyle et al. [8]).

Carbohydrate is predominantly stored as glycogen in both the liver (approximately 100 g) and muscle (approximately 400 g) with 5 g also circulating in the blood stream as glucose. In skeletal muscle, glycogen is typically expressed as mmol·kg^−1^ of dry muscle (d.w.) where concentrations in whole muscle homogenate can vary from 50 to 800 mmol·kg^−1^ d.w., depending on training status, fatigue status and dietary CHO intake (see Figure 1). Muscle glycogen can also be expressed as mmol·kg^−1^ of wet muscle (ww), as commonly reported in the early classical literature, where values range between 10 to 180 mmol·kg^−1^ ww [3,4,5,6].

The glycogen granule itself is essentially a tiered assembly of glucose units (i.e., polymers) that is formed in a branch-like structure via 1:4 and 1:6 α-glycosidic bonds. Glycogen granules are formed on the protein, glycogenin, and can be as large as 42 nm in diameter as well as potentially having 12 tiers. At its maximal size, the granule can consist of as much as 55,000 glucosyl units [18]. Nonetheless, the majority of glycogen granules in human skeletal muscle are reported to be 25 nm in diameter with approximately eight tiers [19]. Although muscle glycogen has traditionally been quantified through acid hydrolysis in whole muscle homogenate, it is, of course, apparent that glycogen is expressed and utilized in fibre type specific patterns as well as being located in specific intracellular locations within muscle cells themselves. Using histochemical techniques, it has typically been reported that resting glycogen content is not apparently different between type I and type II fibres [20,21,22]. Nonetheless, using biochemical quantification (a more quantitative measure), it has been reported that type II fibres may contain 50–100 mmol·kg^−1^ d.w. more glycogen than type I fibres [9,23]. Regardless of method of quantification, glycogen depletion during exercise is dependent on fibre type recruitment patterns, according to the specifics of the exercise protocol. For example, during prolonged steady state type protocols, type I fibres show preferential depletion, whereas during near maximal or supra-maximal type activity, type II fibres become recruited and show considerable glycogen depletion [24]. In activities involving high-intensity intermittent exercise (e.g., a soccer match), considerable glycogen depletion is observed in both muscle fibre types (54% and 46% of type I and II fibres classed as completely or almost empty, respectively) thus reflecting recruitment patterns to support both moderate and high-intensity running speeds [25].

The use of transmission electron microscopy (TEM) has also revealed that glycogen is stored in three distinct sub-cellular pools contained in the myofibrils (intra-myofibrillar glycogen, 5–15% of total glycogen pool), between myofibrils (inter-myofibrallar glycogen, 75% of total glycogen pool) and beneath the sarcolemmal region (sub-sarcolemmal glycogen, 5–15% of total glycogen pool). In endurance trained athletes, it appears that both intra-myofibrillar and sub-sarcolemmal glycogen stores are greater in type I fibres compared with type II fibres, whereas inter-myofibrillar glycogen storage is greater in type II fibres [26]. In relation to acute exercise itself, it is also apparent that intra-myofibrillar glycogen stores show preferential depletion [27], and the failure to restore this specific pool in the immediate hours after exercise is associated with impaired Ca^2+^ release from the sarcoplasmic reticulum [28,29]. Clearly, our understanding of muscle glycogen storage has advanced considerably in recent years, and there remains a definitive need to further quantify intracellular glycogen utilization in a variety of exercise settings, according to training status, age and gender.

## 3. Regulation of CHO Metabolism

**Figure 2:**
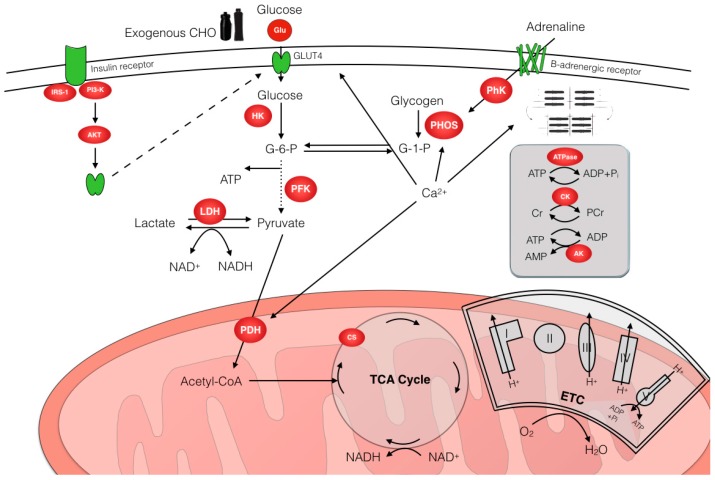
Overview of CHO metabolism and main control points. Key regulatory enzymes are well recognized as phosphorylase (PHOS), hexokinase (HK), phosphofructokinase (PFK), lactate dehydrogenase (LDH) and pyruvate dehydrogenase (PDH). Additionally, the rate of muscle glucose uptake can also determine the flux through glycolysis. Abbreviations: ADP, Adenosine diphosphate; AK, Adenylate kinase; AKT, Protein kinase B; AMP, Adenosine monophosphate; ATP, Adenosine triphosphate; Ca^2+^, Calcium; CHO, Carbohydrate; CK, Creatine kinase; Cr, Creatine; CS, Citrate synthase; ETC, Electron transport chain; G-1-P, Glucose-1-phosphate; G-6-P, Glucose-6-phosphate; Glu, Gluose; GLUT4, Glucose transporter 4; H^+^, Hydrogen ion; H_2_O, water; IRS-1, Insulin receptor substrate 1; HK, Hexokinase; LDH, Lactate dehydrogenase; O_2_, Oxygen; NAD, Nicotinamide adenine dinucleotide; TCA cycle, Tricarboxylic acid cycle; P_i_, phosphate; PCr, Phosphocreatine; PFK, Phosphofructokinase; PhK, Phosphorylase kinase; PHOS, Glycogen phosphorylase; PI3-K, Phosphoinositide 3-kinase.

There are a number of potential sites of control that can regulate the interaction of CHO and lipid metabolism during endurance exercise (see Figure 2). These include the availability of intra-muscular and extra-muscular substrate (controlled by diet and the action of key hormones such as the catecholamines and insulin), the abundance of transport proteins involved in transporting substrates across both the plasma and mitochondrial membranes and, of course, the activity of the key regulatory enzymes involved in the metabolic pathways. The activity of regulatory enzymes can be modified acutely through covalent modification (i.e., phosphorylation and dephosphorylation, largely under hormonal control) and/or allosteric regulation via important signalling molecules that are produced in the muscle as a result of contraction (e.g., adenosine diphosphate (ADP), adenosine monophosphate (AMP), inosine monophosphate (IMP), inorganic phosphate (Pi), calcium (Ca^2+^), hydrogen ion (H^+^). Enzyme activity can also be modified through substrate activation or product inhibition such that increasing the substrate concentration increases catalysis, whereas increased product concentration may inhibit the reaction. Finally, enzyme activity can be regulated chronically through increasing the muscle cell’s content of the actual enzyme protein (i.e., more of the enzyme is actually present) as would occur with endurance training. Clearly, muscle cells possess a highly coordinated and regulatory network of signalling and feedback pathways which function to ensure ATP demand is matched by ATP synthesis. From a physiological perspective, key factors, such as exercise intensity, duration, nutritional status, training status etc., can all regulate substrate utilization during exercise, largely through influencing the potential regulatory control points discussed above. This section will outline the regulation of CHO utilization during endurance exercise, where we pay particular attention to what are currently considered the predominant sites of regulation that are relevant to the specific situation. 

### 3.1. Effects of Exercise Intensity and Duration

As exercise intensity progresses from moderate (i.e., 65% VO_2max_) to high-intensity (85% VO_2max_), muscle glycogenolysis, liver glycogenolysis and glucose uptake increase such that CHO metabolism predominates [33,34]. In contrast, there is a reduction in whole body lipid oxidation due to a reduction in both plasma free fatty acids (FFA) and intramuscular triglyceride oxidation. Maximal rates of lipid oxidation are considered to occur around 65% VO_2max_, though this is dependent on a number of other factors, such as training status, gender and diet [35].

The breakdown of muscle glycogen to glucose-1-phosphate is under the control of glycogen phosphorylase, and this reaction requires both glycogen and Pi as substrates. Phosphorylase, in turn, exists as a more active *a* form (which is under the control of phosphorylation by phosphorylase kinase) and also as a more inactive *b* form (which exists in a dephosphorylated form due to the action of protein phosphatase 1). Given that phosphorylase can be transformed via covalent modification (i.e., phosphorylation by phosphorylase kinase) mediated through epinephrine, it would be reasonable to expect that greater phosphorylase transformation from *b* to *a* may be one mechanism to explain the increased glycogenolysis evident with increasing exercise intensity. This would also be logical given that sarcoplasmic Ca^2+^ levels would be increased with high-intensity exercise (given the need for more rapid cross-bridge cycling) and that Ca^2+^ is a potent positive allosteric regulator of phosphorylase kinase through binding to the calmodulin subunit [36]. However, the percentage of phosphorylase in the more active *a* form does not appear to be increased with exercise intensity and, in actual fact, is decreased after only 10 min of high intensity exercise, which may be related to the reduced pH associated with intense exercise [37]. Whereas this mechanism of transformation (mediated by Ca^2+^ signalling) may be in operation within seconds of the onset of contraction [38], it appears that post-transformational mechanisms are in operation during more prolonged periods of high-intensity exercise given that glycogenolysis still occurs despite reduced transformation. In this regard, vital signals related to the energy status of the cell play more prominent roles. Indeed, as exercise intensity progresses from moderate to high-intensity exercise, the rate of ATP hydrolysis increases so much that there is a greater accumulation of ADP, AMP and Pi. In this way, the increased accumulation of Pi as a result of increased ATP hydrolysis can increase glycogenolysis, as it provides the increased substrate required for the reaction. Furthermore, greater accumulations of free ADP and AMP can also subsequently fine-tune the activity of phosphorylase *a* through allosteric regulation [37]. 

In addition to muscle glycogen, the contribution of plasma glucose to ATP production also increases with exercise intensity. The most likely explanation for this is due to increased muscle blood flow (and hence substrate delivery) in addition to increased muscle fibre recruitment [39]. The delivery of glucose to the contracting muscle is, of course, also a reflection of increased rates of liver glycogenolysis in accordance with increases in exercise intensity [40]. The regulation of liver metabolism during exercise is beyond the scope of the present review, and the reader is directed to the comprehensive review by Gonzalez et al. [34]. Although glucose uptake is also regulated by GLUT4 content, GLUT4 is unlikely to play a role in this situation given that GLUT4 translocation to the plasma membrane is not increased with exercise intensity [41]. Once glucose is transported into the cytosol, it is phosphorylated to glucose-6-phosphate under the control of hexokinase. Evidence suggests that hexokinase activity is also not limiting given that patients with type 2 diabetes (who have reduced maximal hexokinase activity) display normal patterns of exercise-induced glucose uptake likely due to normal perfusion and GLUT4 translocation [42]. In contrast, during intense exercise at near maximal or supra-maximal intensity, glucose phosphorylation may be rate limiting to glucose utilization given that high rates of glucose-6-phosphate, secondary to muscle glycogen breakdown, can directly inhibit hexokinase activity [43]. Once glucose enters the glycolytic pathway, the rate limiting enzyme to glycolysis is considered to be phosphofructokinase (PFK). PFK is allosterically activated by ADP, AMP and Pi, and this mechanism is likely to explain high rates of glycolysis during intense exercise even in the face of metabolic acidosis when PFK could be inhibited. 

In contrast to high intensity exercise, prolonged steady state exercise lasting several hours is characterized by a shift towards increased lipid oxidation and reduced CHO oxidation rates [44]. This shift in oxidation rates is accompanied by an increased contribution of plasma FFA towards energy expenditure and a decreased reliance on both muscle glycogen and intramuscular triglycerides (IMTGs) [44]. Studies examining the regulatory mechanisms underpinning this shift in substrate utilization have suggested that a reduction in muscle glycogen availability (due to progressive glycogen depletion and hence a reduced glycolytic flux) down-regulate pyruvate dehydrogenase (PDH) activity, thereby leading to reduced CHO oxidation. In addition, progressive increases in plasma FFA availability (due to continual lipolysis in adipose tissue) stimulate lipid oxidation. The down-regulation of PDH activity as exercise duration progresses may be due to reduced pyruvate flux, therefore, reducing the substrate production required for the PDH reaction [44]. In addition, more recent data demonstrate an up-regulation of PDH kinase activity during exercise which would, therefore, directly inhibit PDH activity [45]. Taken together, these data are consistent with the many observations that increasing or decreasing substrate availability is one of the most potent regulators of fuel utilization patterns during exercise, and this concept is discussed in the next section.

### 3.2. Effects of Substrate Availability

Modifying substrate availability through dietary manipulation (such as loading regimens, pre-exercise meals or providing enhanced substrate availability during exercise) has been consistently shown to alter metabolic regulation during endurance exercise through various control points. Increasing muscle glycogen concentration enhances glycogenolysis during exercise [46] by enhancing phosphorylase activity given that glycogen is a substrate for phosphorylase. The enhanced glycogenolysis with elevated glycogen stores does not appear to affect muscle glucose uptake [46,47]. In addition to glycogenolysis, muscle glycogen also appears to be a potent regulator of PDH activity (and thus CHO oxidation) during exercise. Indeed, commencing exercise with reduced muscle glycogen attenuates the exercise-induced increase in PDH activity and vice versa [48], likely due to reduced glycolytic flux as well as an increased resting content of pyruvate dehydrogenase kinase 4 (PDK4, the kinase responsible for deactivating PDH) when glycogen concentrations are low. PDH regulation appears particularly sensitive to nutritional status, even at rest. In fact, just 3 days of a low CHO (but increased fat) diet up-regulates PDH kinase activity and down-regulates PDH activity [49]. 

Although the effects of exercise intensity on substrate utilization were discussed previously, it appears that muscle glycogen availability can influence fuel metabolism over and above that of exercise intensity. Indeed, Arkinstall et al. [47] observed that glycogen utilization was enhanced during exercise at 45% VO_2max_ that was commenced with high glycogen (591 mmol·kg^−1^ d.w.) as opposed to exercise at 70% VO_2max_ commenced with low glycogen concentration (223 mmol·kg^−1^ d.w.), despite the higher intensity. In contrast to glycogen utilization and CHO oxidation rates, lipid oxidation was highest when exercise was commenced with reduced glycogen stores. The shift towards fat oxidation when pre-exercise muscle glycogen is low is likely mediated by a number of contributing factors. Firstly, a reduced glycogen availability is associated with an increased epinephrine concentration and plasma FFA availability, thus favoring conditions for augmented lipolysis and lipid oxidation, respectively, compared with conditions of high glycogen concentration [47]. However, when a pre-exercise meal is ingested, and glucose is infused during glycogen depleted exercise such that minimal differences exist between plasma FFA and epinephrine, lipid oxidation is still augmented [50]. In such circumstances, available evidence points to regulation within the muscle cell itself and more specifically, a carnitine mediated increase in lipid oxidation. Carnitine is important given its role as a substrate for carnitine palmitoyltransferase 1 (CPT-1) activity, the rate limiting enzyme that facilitates long chain fatty acid entry to the mitochondria. Indeed, these researchers observed lower PDH activity and acetyl CoA and acetyl carnitine content, and an increased free carnitine concentration during glycogen depleted exercise when compared with glycogen loaded conditions. Interestingly, acetyl CoA carboxylase (ACC) phosphorylation increased and malonyl CoA decreased similarly in both conditions, despite higher AMP activated protein kinase (AMPK) activity when glycogen was reduced. Such data provide further support for a critical role of carnitine in regulating the interaction between CHO and lipid utilization [51] and suggest that malonyl CoA (as an allosteric inhibitor of CPT-1 activity) does not play a major role in fine-tuning fat oxidation in human skeletal muscle. Nonetheless, it is acknowledged that the apparent disconnect between malonyl CoA, CPT-1 activity and in vivo lipid oxidation may be dependent on palmitoyl-CoA concentrations [52]. 

In addition to muscle glycogen availability, consuming CHO in a pre-exercise meal and/or during exercise also induces potent effects on the regulation of CHO and lipid metabolism during exercise. Indeed, CHO ingestion during exercise appears to suppress or even abolish hepatic glucose output during exercise, thus attenuating the decline in liver glycogen content [53]. In relation to lipid metabolism, CHO feeding attenuates lipolysis in adipose tissue (as mediated via anti-lipolytic effects of insulin) such that plasma FFA availability is reduced during exercise undertaken in CHO fed conditions [54]. Horowitz et al. [54] studied male participants during 60 min of exercise at 45% VO_2max_ in fasted conditions, or 1 h after consuming 0.8 g·kg^−1^ of glucose (to induce a high insulin response) or 0.8 g·kg^−1^ fructose (to induce a low insulin response), or in an additional glucose trial during which intralipid and heparin were infused so as to maintain plasma FFA availability in the face of high insulin. In accordance with the insulin response in CHO fed conditions, lipolysis (as indicated by rate of appearance of glycerol), FFA availability and lipid oxidation were reduced. However, when intralipid and heparin were infused during the additional glucose trial, lipid oxidation rates were enhanced by 30% (4.0 µmol·kg^−1^·min^−1^) compared with the glucose only trial (3.1 µmol·kg^−1^·min^−1^) but were still not restored to levels occurring during fasted exercise (6.1 µmol·kg^−1^·min^−1^). Taken together, whilst these data suggest that only small elevations in insulin can attenuate lipolysis (i.e., 10–30 µU/mL), they also demonstrate a limitation within the muscle cell itself during CHO fed conditions. 

In an effort to ascertain the source of limitation to lipid oxidation within the muscle following CHO feeding, Coyle et al. [55] infused octanoate (a medium chain fatty acid, MCFA) or palmitate (a long chain fatty acid, LCFA) during 40 min of exercise at 50% VO_2max_ after an overnight fast or 60 min after ingesting 1.4 g·kg^−1^ of glucose. As expected (based on the previously discussed study), plasma FFA and lipid oxidation were higher in the fasted trials whilst CHO oxidation was lower compared with the glucose trials. However, the major finding of this study was that the percentage of palmitate oxidized during the glucose trial was reduced compared with fasting (70% vs. 86%, respectively) whereas octanoate was unaffected (99% vs. 98%, respectively). These data, therefore, suggest that LCFA uptake into the mitochondria is reduced with CHO feeding. When taken in the context of previous sections, it becomes apparent that any condition which accelerates glycolytic flux (e.g., increased intensity, muscle glycogen, glucose feeding) can down-regulate intramuscular lipid metabolism. Furthermore, the increased insulin and decreased epinephrine levels which accompany glucose ingestion during exercise appear to result in the attenuation of intra-muscular hormone sensitive lipase (HSL) activity [56], thus highlighting an additional point of control.

### 3.3. Effects of Training Status

Endurance training results in a number of profound physiological and metabolic adaptations which function to reduce the degree of perturbations to homeostasis for a given exercise intensity and ultimately, delay the onset of fatigue. Adaptations to endurance training are most recognized functionally by an increase in maximal oxygen uptake as well as a rightward shift in the lactate threshold. From a metabolic perspective, the most prominent adaptation is an increase in the size and number of mitochondria (i.e., mitochondrial biogenesis), which essentially permits a closer matching between ATP requirements and production via oxidative metabolism. The adaptive response of muscle mitochondria is also accompanied by increases in capillary density, substrate transport proteins and increased activity of the enzymes involved in the main metabolic pathways. In addition, endurance training increases the capacity for skeletal muscle, but not liver [34], to store glycogen and triglycerides, thereby increasing substrate availability. In relation to substrate utilization during exercise following endurance training, the most notable response is a reduction in CHO utilization with a concomitant increase in lipid oxidation [57]. 

For a given exercise intensity, glycogen utilization is reduced with exercise training [58], an effect that is confined locally to the actual muscles that were trained [59]. The reduced glycogenolysis observed after training is not due to any change in phosphorylation transformation but rather, allosteric mechanisms [60,61]. Indeed, exercise in the trained state is associated with reduced contents of ADP, AMP and Pi, thereby providing a mechanism leading to reduced phosphorylase activity. Le Blanc et al. [61] also observed reduced pyruvate and lactate production during exercise undertaken in the trained state as well as reduced PDH activity. As a result of the reduced CHO flux, it is, therefore, likely that the attenuated pyruvate production (in addition to reduced ADP accumulation) may have attenuated PDH activity. In addition to training-induced reductions in muscle glycogenolysis, several investigators have observed that training reduces exercise-induced liver glycogenolysis [34]. In this way, trained individuals will have more liver glycogen available late in exercise to thereby maintain the plasma glucose concentration and hence, support maintenance of the desired exercise intensity. There is some evidence that endurance training also increases gluconeogenesis following training [62]. In accordance with reduced rates of glucose production, muscle glucose uptake is reduced when exercise is undertaken at the same absolute workload following a period of endurance training [63].

Despite the fact that training increases total muscle GLUT4, the reduction in exercise-induced muscle glucose uptake is likely caused by a reduced translocation of GLUT4 to the sarcolemma following training, thereby reducing the capacity to transport glucose [64]. One particular study utilized a knee extensor training and exercise model where only one limb was trained but yet both limbs performed the exercise protocol before and after training. In this way, training-induced alterations in hormonal and cardiovascular status were minimized and the reduced glucose uptake and GLUT4 translocation were likely mediated by local contractile factors. In summarizing the link between liver glucose production and muscle glucose uptake, it is generally accepted that training-induced changes in hormone concentrations such as epinephrine, insulin and glucagon are unable to explain all of the effects [65]. Rather, it is possible that the actual rate of muscle glucose uptake acts as a feedback signal to regulate glucose output from the liver [65].

## 4. CHO and Exercise Performance

Given the effects of exercise intensity, duration and training status on muscle glycogen utilization, it follows that glycogen depletion (in both muscle and liver) is a major cause of fatigue in both endurance and high-intensity (intermittent) type activities. As such, traditional nutritional advice for these types of activities (whether it is competitive situations or training sessions) is to ensure high daily CHO intake before, during and after the activity so as to promote both performance and recovery. 

### 4.1. Muscle Glycogen and CHO Loading

The basic principles of CHO loading were developed in the late 1960s where it was identified that a period of exhaustive exercise followed by several days of high dietary CHO intake induces a super-compensation effect so that glycogen storage is augmented [3,4]. A less extreme form of CHO loading was developed in the 1980s where Sherman et al. [66] observed that a simple exercise taper in conjunction with several days of increased dietary CHO intake was also sufficient to increase glycogen storage. It is now generally accepted that trained athletes can increase glycogen storage in both type I and II fibres within 24–48 h of increased CHO intake [67]. In relation to practical application, it is also suggested that high glycemic foods are superior to low glycemic foods [68] in augmenting glycogen storage and that dietary intakes of 8–12 g·kg^−1^ per day are likely required to “maximize” glycogen storage [7]. The general consensus from the wealth of studies undertaken in the last 40 years is that CHO loading can improve performance and capacity when the exercise is greater than 90 min in duration [69]. The enhanced performance effect is likely initially mediated by a delay in the time-point at which energy availability becomes limiting to the maintenance of the desired workload, which, in the case of race pace, is dependent on sustained and high rates of CHO oxidation [70,71]. Indeed, in reviewing the literature, Hawley et al. [69] cited that CHO loading can improve exercise capacity by approximately 20%, and time trial performance can increase by 2–3%. In addition to providing substrate availability for ATP production, it is now recognized that glycogen availability (especially the intramyofibrillar storage pool) can directly modulate contractile function. Indeed, a series of studies from Ørtenblad and colleagues [28,29,72] have collectively shown preferential utilization of this storage pool during exercise in a manner that also correlates with impaired Ca^2+^ release from the sarcoplasmic reticulum. Such impaired excitation–contraction coupling is likely to be of particular importance during situations where higher power outputs and sprint finishes are required in the very late and finishing stages of races. 

### 4.2. Pre-Exercise CHO Availability

Whereas the 1960s and 1970s focused on CHO loading studies, research in the next two decades examined the effects of pre-exercise feeding as well as consuming additional CHO during exercise. Pre-exercise feeding (i.e., 3–4 h before competition) is not only advantageous as it can lead to further elevations in muscle glycogen content [73] but can also restore liver glycogen content, which is usually depleted after an overnight fast. The latter is particularly important given that liver glycogen content is also related to exercise capacity [13]. Sherman et al. [74] observed that time trial performance after 90 min of steady state exercise at 70% VO_2max_ was greater when 150 g of CHO was consumed before exercise compared with 75 g of CHO, both of which were greater than no meal. The enhanced performance effect was associated with the maintenance of blood glucose concentration late into exercise, which is important because liver glucose production and muscle glucose uptake and oxidation become more important when muscle glycogen concentrations begin to decline. In a further study, the same authors also observed that performance can be further increased when CHO is ingested during exercise in addition to a pre-exercise meal [75]. As such, current CHO guidelines for pre-exercise feeding advise an intake of 1–4 g·kg^−1^ body mass, 3–4 h prior to exercise [7].

### 4.3. CHO Feeding during Exercise

In addition to high endogenous pre-exercise muscle and liver glycogen stores, it is widely accepted that exogenous CHO feeding during exercise also improves physical elements of performance [76]. Whereas it was generally accepted that exogenous CHO oxidation rates were limited at approximately 1 g·min^−1^ due to saturation of intestinal glucose transporters, it is now known that exogenous CHO oxidation rates can increase to 1.8 g·min^−1^ with the addition of sucrose or fructose to the CHO blend [77]. When taken together, it is currently thought that CHO feeding during exercise may, therefore, augment exercise performance via multiple mechanisms, consisting of muscle glycogen sparing [22], liver glycogen sparing [53] and maintenance of plasma glucose and CHO oxidation rates [8]. The role of CHO feeding in reducing liver glycogen breakdown as a performance enhancing mechanism is gaining increasing recognition [78]. In simple terms, a liver glycogen sparing effect ensures that more liver glycogen is, therefore, available late in exercise, thereby maintaining plasma glucose availability and delivery to the muscle to meet the CHO oxidation rates necessary to sustain the required workload. 

It is noteworthy that exogenous CHO feeding during exercise also improves performance [11] when exercise duration is <60 min (i.e., where muscle and liver glycogen availability is not likely limiting), an effect that is not apparent when glucose is directly infused to the bloodstream during exercise [79]. Such data suggest that CHO feeding may also improve exercise performance via non-metabolic effects but through direct effects on the central nervous system [14]. To this end, the last decade of research has resulted in a growing body of literature demonstrating that simply “rinsing” CHO in the oral cavity (for 10-s periods every 5–10 min during exercise) is also ergogenic to performance [80], an effect that is independent of sweetness [15] and that is especially apparent in the absence of a pre-exercise CHO meal [81] and low pre-exercise muscle glycogen [82], although this effect is not always evident [83].

The conventional approach to CHO fueling during exercise is to consume 6–8% CHO beverages, although relying solely on this approach does not allow for flexibility in terms of individual variations in body mass or actual fluid requirements given variations in ambient conditions [84]. As such, many athletes rely on a CHO fueling approach that is based on a combination of solids (e.g., bars), semi-solids (e.g., gels) and fluids (e.g., sports drinks) so as to collectively meet their personalized exogenous CHO targets, typically in the region of 30–90 g·h^−1^, depending on exercise duration. Nevertheless, although there is little difference in exogenous CHO oxidation rates (albeit in fluid matched conditions) between the aforementioned sources [85,86], it is noteworthy that many athletes experience gastrointestinal discomfort when attempting to hit these targets, possibly related to extreme differences in osmolality between commercially available CHO gels [87] as well as the presence of fibre, fat and protein in energy bars [88]. As such, it is now advised that athletes should clearly practice their approach to in-competition fueling during training sessions of a similar intensity and duration as competition. As a general rule of thumb, it is suggested that 30–60 g·h^−1^ of CHO (glucose polymers e.g., maltodextrin) is consumed during events lasting < 60–150 min [78], whereas in events > 2.5–3 h, 60–90 g·h^−1^ (glucose/fructose blends) is the recommended rate [7]. Whilst beyond the scope of the present review, it is noteworthy that CHO ingestion (in either drink or gel format) during team sport type activity (i.e., <90 min duration) can also improve performance of technical skills (see reference [89] for an extensive review on this topic), thus providing further evidence for the ergogenic properties of CHO feeding during exercise.

## 5. CHO and Training Adaptations

### 5.1. Overview of Molecular Regulation of Training Adaptations

**Figure 3:**
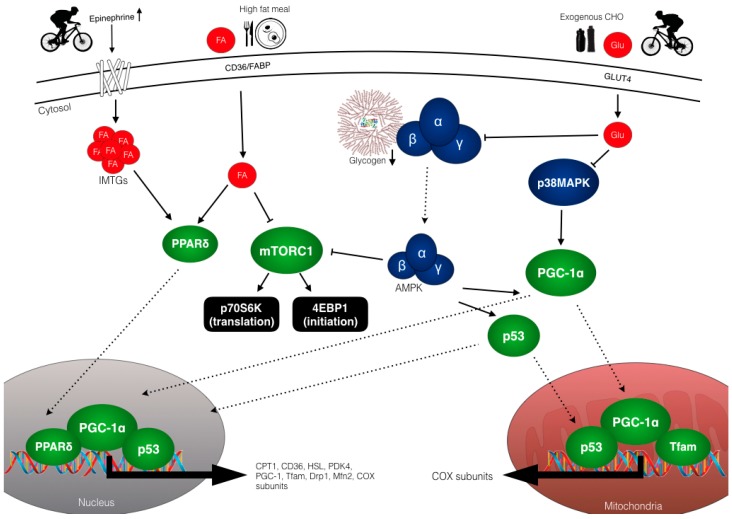
Schematic overview of the potential exercise-nutrient sensitive cell signalling pathways regulating the enhanced mitochondrial adaptations associated with training with low CHO availability. Reduced muscle glycogen and exogenous glucose availability enhance both AMPK and p38MAPK phosphorylation which results in activation and translocation of PGC-1α and p53 to the mitochondria and nucleus. Upon entry into the nucleus, PGC-1α co-activates additional transcription factors, (i.e., NRF1/2) to increase the expression of COX subunits and Tfam as well as auto-regulating its own expression. In the mitochondria, PGC-1α co-activates Tfam to coordinate the regulation of mtDNA and induces expression of key mitochondrial proteins of the electron transport chain, e.g., COX subunits. Similar to PGC-1α, p53 also translocates to the mitochondria to modulate Tfam activity and mtDNA expression and to the nucleus where it functions to increase the expression of proteins involved in mitochondrial fission and fusion (DRP-1 and MFN-2) and electron transport chain protein proteins. Exercising in conditions of reduced CHO availability increases adipose tissue and intramuscular lipolysis via increased circulating epinephrine concentrations. The resulting elevation in FFA activates the nuclear transcription factor, PPARδ, to increase expression of proteins involved in lipid metabolism, such as CPT-1, PDK4, CD36 and HSL. However, consuming pre-exercise meals rich in CHO and/or CHO during exercise can down-regulate lipolysis (thereby negating FFA mediated signalling) as well as reducing both AMPK and p38MAPK activity, thus having negative implications for downstream regulators. High fat feeding can also modulate PPARδ signalling and up-regulate genes with regulatory roles in lipid metabolism (and down-regulate CHO metabolism), though high fat diets may also reduce muscle protein synthesis via impaired mTOR-p70S6K signalling, despite feeding leucine rich protein. Abbreviations: 4EBP1; eukaryotic translation initiation factor 4E-binding protein 1, AMPK; AMP-activated protein kinase, CHO; carbohydrate, COX; cytochrome c oxidase, CPT-1; carnitine palmitoyltransferase 1, Drp1; dynamin-related protein 1, FA; fatty acid, FABP; fatty acid binding protein, GLU; glucose, HSL; hormone sensitive lipase, IMTG; intramuscular triglycerides, Mfn2; mitofusion-2, mTORC1; mammalian target of rapamycin complex 1, p38 mitogen-activated protein kinase, p70S6K; ribosomal protein S6 kinase, PDK4; pyruvate dehydrogenase kinase 4, PGC-1α; peroxisome proliferator-activated receptor gamma coactivator 1-alpha, PPARδ; peroxisome proliferator-activated receptor, Tfam; mitochondrial transcription factor A.

Skeletal muscle is a highly malleable tissue that has the ability to undergo major adaptations and alter its phenotype in response to exercise stimuli. In relation to endurance training, prominent adaptations include increased mitochondrial biogenesis, lipid oxidation and angiogenesis, recognized functionally by a rightward shift of the lactate threshold curve [90]. Upon the onset of muscle contraction, the accumulation of multiple metabolic signals generated during exercise (i.e., increased AMP/ATP ratio, Ca^2+^ flux, lactate, hypoxia and energy availability) initiates a cascade of events that activate or suppress specific signalling pathways that regulate gene expression and protein translation [91,92,93,94]. The dynamic fluctuation in content and subcellular location of metabolites activates regulatory cell signalling kinases that converge on nuclear and mitochondrial transcription factors, and co-activators to induce a co-ordinated up-regulation of both nuclear and mitochondrial genomes [95]. It is the combination of transient increases in gene expression and protein content that ultimately form the molecular basis of training adaptations. Many exercise regulated signalling pathways are also sensitive to nutrient availability [17], and a schematic overview of the potential exercise and nutrient interactions that modify the early signalling events regulating mitochondrial biogenesis is displayed in Figure 3.

As previously discussed, the principle of promoting high CHO availability before, during and after exercise is the foundation on which traditional sports nutrition guidelines are based. Although this is essential for promoting competition performance and ensuring adequate recovery, accumulating data now suggest that restricting CHO before, during, and in recovery from endurance-based exercise augments the cell signaling and gene expression responses associated with oxidative adaptations in human skeletal muscle. Indeed, both acute and training based studies have collectively observed that the reduction of both endogenous and/or exogenous CHO promotes an increase in mitochondrial enzyme activity and protein content, increases both whole body [96] and intramuscular [97] lipid metabolism and can improve both exercise capacity [98] and performance [99]. This approach to CHO periodization has been termed “train-low, compete-high”, a model which promotes carefully scheduled periods of CHO restricted training for augmenting adaptation, but ensures high CHO availability prior to and during competition in order to promote maximal performance.

### 5.2. Low Muscle Glycogen Availability and Twice per Day Training Models

The altered metabolic milieu created through exercising with low glycogen availability has a direct impact on molecular signalling events controlling muscular adaptations. The notion that CHO restriction augments markers of training adaptation emerged from an initial investigation from Pilegaard et al. [100] who observed an enhanced expression of genes involved in mitochondrial biogenesis and substrate utilization when exercise was undertaken with reduced muscle glycogen. Indeed, exercise induced PDK4 and UCP3 gene expression were both augmented with low (240 ± 38 mmol·kg^−1^ d.w.) pre-exercise muscle glycogen when compared to normal (398 ± 52 mmol·kg^−1^ d.w.) glycogen levels. On the basis of the molecular evidence derived from acute studies, initial training studies adopted a “training twice every second day versus once daily” model. Using this model, Hansen et al. [98] subjected seven untrained males to 10 weeks of knee extensor exercise training under conditions of either high or low muscle glycogen. Subjects trained both legs according to two different schedules, whereby one leg was trained every day (HIGH) whilst the contralateral leg was trained twice a day, every other day (LOW). Exercise during the twice per day sessions was interspersed with 2 h of recovery, during which time CHO intake was restricted. As such, one leg (LOW) commenced 50% of training sessions under conditions of low glycogen, while the other leg performed each session under conditions of high muscle glycogen (HIGH), whilst allowing the matching of total work done between legs. Following 10 weeks of training, the leg that commenced 50% of training sessions with low muscle glycogen demonstrated a superior increase in the maximal activity of both citrate synthase (CS) and β-hydroxyacyl-CoA dehydrogenase (β-HAD) when compared with the contralateral leg. Furthermore, the LOW leg also demonstrated an almost two-fold increase in exercise capacity when compared with the HIGH leg, thus demonstrating a potent effect of altering substrate availability on exercise-induced adaptation and subsequent performance. 

Yeo and colleagues [96] subsequently adopted a “real world” design more applicable to elite athletes. Using well trained male cyclists completing a 3-week training block, cyclists trained six times per week, either once every day with high muscle glycogen availability or twice every other day, so the second session was undertaken with reduced levels of muscle glycogen. In the “high” group, cyclists alternated between steady-state and high-intensity training (HIT) each day, whereas in the “low” group, steady-state exercise was performed in the morning and HIT exercise was performed after a 1–2 h recovery period during which time CHO was restricted. Before and after this training block, muscle biopsies were obtained to assess markers of adaptation, and a time trial was completed to examine performance improvements in each group. Despite significant increases in CS and β-HAD activity, cytochrome c oxidase subunit IV (COXIV) protein content and rates of fat oxidation in the “low” group following training, training-induced improvements in time trial performance were comparable between groups. Interestingly, the enhanced adaptive responses occurred in the “low” group despite cyclists having to reduce exercise intensity during the HIT training session. These findings suggest that even when overall training intensity is reduced, reduced CHO availability is associated with an adaptive response. In a similar study design, Hulston and colleagues [97] reported greater increases in intra-muscular lipid oxidation and the expression of CD36 and β-HAD activity following “training low” compared with “training high”. 

### 5.3. Fasted Training

Performing endurance training in the fasted state represents a simpler model of “training low” where exercise is performed prior to breakfast. Although pre-exercise muscle glycogen is not altered as a result of the overnight fast, liver glycogen remains lower whilst FFA availability is increased [54] compared with when breakfast is fed. Exercising in the fasted state increases post-exercise AMPK activity [101] and mRNA of genes controlling substrate utilization (PDK4, GLUT4, CD36, CPT-1) and mitochondrial function (UCP3) [102,103] compared with when CHO is fed before and during exercise. Accordingly, chronic periods of fasted training elicit similar adaptations to those observed when training with low muscle glycogen. Nybo et al. [104] demonstrated that 8 weeks of endurance training (50–90 min of high-intensity intervals at 70–85% VO_2max_) in the fasted state enhanced training induced increases in β-HAD activity and basal muscle glycogen content compared with when CHO was fed before and during training. Similarly, Van Proeyen et al. [105] observed augmented CS and β-HAD activity when regular steady state (1–1.5 h cycling at 70% VO_2max_) cycling was performed in the fasted state compared to when breakfast was fed. Nonetheless, the augmented biochemical adaptations did not translate to improved exercise performance. 

### 5.4. Post-Exercise CHO Restriction

In addition to restricting CHO prior to endurance exercise training, data also demonstrate beneficial adaptive responses when restricting CHO during the post-exercise recovery period. Indeed, Pilegaard et al. [106] explored this idea with participants completing 75 min of cycling at 75% VO_2max_ followed by the consumption of a diet either high or low in CHO for the next 24 h. These authors observed that although the mRNA expression of pyruvate dehydrogenase kinase 4 (PDK4), lipoprotein lipase (LPL), uncoupling protein 3 (UCP3), and carnitine palmitoyltransferase 1 (CPT1) increased in response to exercise, expression levels were sustained at 24 h post-exercise in the low CHO group only. In a twice per day, 6-week training study, it was also observed that when glucose is consumed during recovery from the first session, the enhanced oxidative adaptations are blunted compared to when CHO is restricted, despite reduced levels of muscle glycogen [107]. When taken together, data from these studies suggest that reducing CHO availability in the recovery period also modulates the muscle adaptive process. 

### 5.5. Sleep-Low, Train-Low Models

More recent train-low investigations have adopted a “sleep-low, train-low” model, whereby participants perform an evening training session, restrict CHO overnight and complete a training session the subsequent morning under levels of low muscle glycogen availability. Acute studies using whole body exercise [31] demonstrate that commencing HIT running with low muscle glycogen (as a result of glycogen depleting exercise the prior evening) leads to significant phosphorylation of both ACC and p53 alongside enhanced gene expression of PGC-1α, COXIV, Tfam and PDK4. In contrast, when exercise was commenced with high muscle glycogen and exogenous CHO was provided before, during and after exercise, the aforementioned activation of signalling kinases and gene expression were completely abolished. In a subsequent study, Lane et al. [108] manipulated the timing of daily CHO ingestion to elicit sleeping with reduced muscle glycogen. In this way, subjects consumed either 8 g·kg^−1^ CHO prior to a bout of evening HIT (and subsequently restricted CHO intake overnight) or consumed 4 g·kg^−1^ CHO prior to the evening HIT and 4 g·kg^−1^ CHO post-exercise in order to replenish muscle glycogen. Although fat oxidation during morning exercise and post-exercise PDK4 mRNA expression were significantly greater in the sleep-low group, the genes involved in the regulation of mitochondrial biogenesis displayed similar exercise induced increases in both groups [108]. Using a similar sleep-low model as Lane and colleagues [108], Marquet et al. [99] observed that 3 weeks of sleep-low training in elite triathletes and cyclists improved cycling efficiency (3.1%), 20 km cycling time trial performance (3.2%) and 10 km running performance (2.9%) compared with traditional high CHO approaches. 

While the mechanisms underpinning the aforementioned adaptive responses to both acute and chronic exercise are still not fully understood, they are likely mediated by upstream signalling kinases. Indeed, AMPK has the capacity to be modulated by the glycogen status of the muscle through a glycogen-binding domain on the β-subunit [109]. Wojtaszewski et al. [92] demonstrated that when pre-exercise muscle glycogen levels are reduced, AMPKα2 activity and ACC^Ser221^ phosphorylation are significantly elevated following steady state cycling compared to when muscle glycogen is high. In a subsequent study, Chan et al. [110] also observed a significantly greater nuclear abundance of p38MAPK, both pre and post exercise, when muscle glycogen levels were low. In a twice per day, train-low model, Cochran et al. [93] also reported significantly greater elevations in p38MAPK phosphorylation following the second exercise session when participants consumed no CHO during recovery. These data are highly suggestive of both AMPK and p38MAPK being nutrient sensitive, and thus likely regulating the downstream events (e.g., p53 and PGC-1α activation) that co-ordinate mitochondrial biogenesis.

### 5.6. Critical Limitations of Train-Low Models

Despite the theoretical rationale for train-low protocols, it is noteworthy that the augmented cell signalling responses, enzymatic changes and improved performance outcomes are not always apparent or consistent between studies (see Impey et al. [111] for an extensive review of this topic). Indeed, recent work from Gejl et al. [112] observed no additional benefit when elite triathletes performed selected training sessions with low CHO availability on both CS and β-HAD maximal activity when compared with traditional high CHO availability approaches. Similarly, Burke et al. [113] observed that periodising CHO availability (to incorporate train low sessions) offered no additional benefit to 10 km race walking performance in Olympic standard race walkers when compared with high CHO availability. However, given that the enhanced training response associated with train-low is potentially mediated by muscle glycogen availability, close examination of the available glycogen data between studies may explain the lack of molecular, biochemical and performance changes reported within some train-low studies. In this regard, we recently suggested the concept of a glycogen threshold, whereby the beneficial adaptations associated with train-low paradigms are especially apparent when the exercise session is commenced with muscle glycogen concentrations < 300 mmol·kg^−1^ d.w. [111]. To this end, there is, therefore, a definitive need to better understand the exercise and nutrient conditions that truly constitute train-low conditions.

### 5.7. Practical Applications

There are also a number of potential limitations to this type of training that can make it difficult for exercise physiologists and nutritionists to optimally periodise this type of training into an elite athlete’s training schedule. For example, reduced CHO availability impairs acute training intensity [96,97] and hence, if performed long-term, may actually lead to a de-training effect. Additionally, given the role of CHO in preventing immunosuppression, it has been suggested that repeated high intensity training under conditions of low CHO increases susceptibility to illness and infection [114]. Nonetheless, proxy markers of immunity and the incidence of upper respiratory tract infections (URTI) appear to be unaltered by 3 weeks of sleep-low training [115]. Restriction of CHO availability has also been shown to increase muscle protein breakdown [116], an effect that, if performed chronically, may lead to muscle mass loss, especially in conditions of both calorie and CHO restriction. Finally, data also demonstrate a reduced ability to oxidize exogenous CHO following regular training with low CHO, which could lead to a negative effect on competition performance [117]. Taking the above limitations into account, it is important to recognize that training with low CHO availability should be carefully periodised in an athlete’s training programme. In practice, this approach could represent an amalgamation of train-low paradigms and is perhaps best communicated by the principle of “fuel for the work required” [111]. In this way, athletes could strategically reduce CHO availability prior to completing pre-determined training workloads that can be readily performed with reduced CHO availability, thereby inducing a “work-efficient” approach to training [118]. Alternatively, when the goals of the training session are to complete the highest workload possible over more prolonged durations, then adequate CHO should be provided in the 24 h period prior to and during the specific training session. Careful day-to-day periodization in a meal-by-meal manner (as opposed to chronic periods of CHO restriction) is likely to maintain metabolic flexibility and still allow for the completion of high-intensity and prolonged duration workloads on heavy training days, e.g., interval type workouts undertaken above lactate threshold. Intuitively, train-low sessions may be best left to training sessions that are not CHO dependent and in which the intensity and duration of the session are not likely to be compromised by reduced CHO availability, e.g., steady-state type training sessions performed at intensities below the lactate threshold. Clearly, more studies are required to investigate the optimal practical approach for which to integrate periods of train-low into an elite athlete’s training programme.

## 6. Summary and Future Directions

Despite over 100 years of research, carbohydrate metabolism continues to intrigue muscle biologists and exercise scientists. From its early recognition as a simple fuel store, it is now apparent that the glycogen granule regulates many cell-signaling processes related to both health and human performance. Nonetheless, it is clear that many of the original questions posed in our field are still relevant today, though the array of biochemical tools now at our disposal ensure we are better equipped to answer those questions with greater precision. For example, the storage of the glycogen granule in specific intracellular pools remains a highly active research area. As a related point, the magnitude of exercise-induced utilization of specific storage pools remains to be documented using “real-world” exercise protocols that are relevant to both training and competition scenarios. Whilst the specific regulatory control points of CHO metabolism are now well documented, the precise molecular mechanisms underpinning the regulation of CHO transport, storage and utilization are not yet fully known. Finally, the identification of the glycogen granule as a regulator of training adaptation has opened a new field of study that is likely to dominate the applied nature of sport nutrition research in the coming decade. From the early studies from the pioneers in the field (e.g., Krogh, Lindhard, Bergstrom, Saltin, etc.), it is clear that our field remains as exciting as ever.
